# Current Treatments and New, Tentative Therapies for Parkinson’s Disease

**DOI:** 10.3390/pharmaceutics15030770

**Published:** 2023-02-25

**Authors:** Teresa Pardo-Moreno, Victoria García-Morales, Sami Suleiman-Martos, Antonio Rivas-Domínguez, Himan Mohamed-Mohamed, Juan José Ramos-Rodríguez, Lucía Melguizo-Rodríguez, Anabel González-Acedo

**Affiliations:** 1 Primary Health Care, Instituto Nacional de Gestión Sanitaria (INGESA), 51003 Ceuta, Spain; 2 Physiology Area, Department of Biomedicine, Biotechnology and Public Health, Faculty of Medicine, University of Cádiz, Pl. Falla, 9, 11003 Cádiz, Spain; 3 Servicio Andaluz de Salud, Avd. del Sur 11, 18014 Granada, Spain; 4 Department of Celular Biology, University of Seville, 41009 Seville, Spain; 5 Department of Physiology, Faculty of Health Sciences (Ceuta), University of Granada, 51001 Ceuta, Spain; 6 Biomedical Group (BIO277), Department of Nursing, Faculty of Health Sciences, University of Granada, 18016 Granada, Spain; 7 Instituto de Investigación Biosanitaria, Ibs Granada, Av. de Madrid, 15, 18012 Granada, Spain

**Keywords:** alpha-synuclein, Lewy bodies, Parkinson’s disease, therapeutic targets

## Abstract

Parkinson’s disease (PD) is a neurodegenerative pathology, the origin of which is associated with the death of neuronal cells involved in the production of dopamine. The prevalence of PD has increased exponentially. The aim of this review was to describe the novel treatments for PD that are currently under investigation and study and the possible therapeutic targets. The pathophysiology of this disease is based on the formation of alpha-synuclein folds that generate Lewy bodies, which are cytotoxic and reduce dopamine levels. Most pharmacological treatments for PD target alpha-synuclein to reduce the symptoms. These include treatments aimed at reducing the accumulation of alpha-synuclein (epigallocatechin), reducing its clearance via immunotherapy, inhibiting LRRK2, and upregulating cerebrosidase (ambroxol). Parkinson’s disease continues to be a pathology of unknown origin that generates a significant social cost for the patients who suffer from it. Although there is still no definitive cure for this disease at present, there are numerous treatments available aimed at reducing the symptomatology of PD in addition to other therapeutic alternatives that are still under investigation. However, the therapeutic approach to this pathology should include a combination of pharmacological and non-pharmacological strategies to maximise outcomes and improve symptomatological control in these patients. It is therefore necessary to delve deeper into the pathophysiology of the disease in order to improve these treatments and therefore the quality of life of the patients.

## 1. Introduction

Parkinson’s disease (PD) is a neurodegenerative disease caused by the death of a type of neuron that plays a fundamental role in the production of dopamine in the brain [1,2]. PD is the second most common neurodegenerative disease, affecting 0.2% of the global world population, 1% of the population over 60 years old, and up to 4% of the population over 80 years old. It has an exponential growth of prevalence, especially in men [3,4,5,6]. PD patients have a risk of dementia that is 6–8 times higher than that of aged-matched controls, with a long-term prevalence of up to 80% [7]. The cause of PD is unknown, with both inherited and environmental factors believed to play a role. Although its origin is often of unknown aetiology, age remains the main risk factor for PD. Most cases are sporadic in origin, although it is estimated that 5% of people with PD have the disease due to a genetic component. The best-known genetic mutation responsible for PD is parkin. This autosomal recessive mutation is present in 50% of cases of early PD (<45 years) and 15% of cases of late PD [8]. In addition, some environmental factors associated with agriculture, such as exposure to pesticides, herbicides, insecticides, solvents, and fertilisers, have shown significant associations with the subsequent development of PD [9]. On the other hand, some studies claim that the consumption of caffeine, tobacco, or alcohol and good hydration and a rural lifestyle are associated with a lower risk of PD [10].

One of the main obstacles to slowing down the deterioration of PD is that it takes a long time to be diagnosed. Tremors, the most obvious sign, can appear years after the onset of the disease. In fact, it has been postulated that PD may have a preclinical phase with an average duration of 15 years [11]. The diagnosis of the disease is usually derived from the appearance of tremors in the limbs, its best-known symptom. These motor symptoms manifest when 50–70% of nigrostriatal dopaminergic function has been lost. However, in 30–40% of cases the patients have no tremor, which may result in half of PD cases going undiagnosed [12]. Moreover, it is estimated that the number of people affected will double in the next 30 years due to the increasing prevalence in an ageing population [13]. Annual costs per patient treated are EUR 3910 per patient in Europe [14] and USD 4551 in the USA [15]. This figure may change in the coming years due to the introduction of new deep-stimulation therapies, which have high initial costs (32,363 euros) but subsequently reduce annual treatment costs to EUR 1295 per patient [14].

The death of dopaminergic neurons means that PD is characterised by very specific motor symptoms: tremor, rigidity, bradykinesia, inability to initiate voluntary movements, and postural instability. Although these symptoms are the most characteristic, other non-motor features also appear in PD: autonomic dysfunction, mood disorders, sleep disorders, cognitive impairment, gastrointestinal symptoms, and pain [16,17]. The mean age of the onset of symptoms of PD was estimated to be in the late fifties; however, with the increase in the average age, the mean age of symptom onset has risen to the sixties [18,19,20]. All existing treatments are aimed at alleviating symptoms and there is currently no therapeutic treatment to cure PD.

To understand the pathophysiological basis of PD, it is necessary to know the physiology that underlies PD (the neuronal pathways of the SN). For many years, Parkinson’s disease (PD) was viewed as the result of the progressive degeneration of dopaminergic nerve cells in the substantia nigra (SN), causing the key motor symptoms of the disease. However, recent clinical and pathological observations have shown that this only represents a limited aspect of PD. Thus, at present, the term “Parkinson’s disease” comprises three stages: (1) the preclinical phase, when the neurodegenerative process has commenced but is not yet causing any symptoms; (2) the prodromal phase, when the progression of neurodegeneration in various regions of the central nervous system (CNS) and peripheral nervous system (PNS) is causing noticeable symptoms; and (3) the clinical phase, when the classic motor symptoms are prominent enough to diagnose PD. Thus, the first signs of the disease may be present prior to the onset of classic motor symptoms without a substantial loss of dopaminergic neurons in the brain [21]. Lewy bodies are not limited to appearing in the SN and have been observed in the cortex, amygdala, locus coeruleus, vagal nucleus, and peripheral autonomic nervous system. This could be one of the causes of non-motor symptoms. Five main pathways in the brain connect the basal ganglia with other areas of the brain. They are known as the motor, oculomotor, associative, limbic, and orbitofrontal circuit pathways, with their nomenclature indicating the main projection area of each circuit. All these pathways are affected in advanced stages of PD, and their malfunction explains the symptoms of the disease [22]. The motor symptomatology in PD is understood thanks to the fact that the motor control pathways of the basal ganglia are defined. In the striatum (caudate nucleus and putamen), sensory afferent information converges from the cerebral cortex and SN pars compacta. Regarding the efferent, the substantia nigra reticularis and the globus pallidus represent the exit pathway for the basal ganglia. At rest (the absence of voluntary movement), neurons in the reticular SN and globus pallidus are tonically active (inhibiting neurons in the superior colliculus and nuclei of the thalamus). The cells of the thalamus and superior colliculus escape tonic inhibition if the neurons of the reticular SN and globus pallidus are inhibited by the GABAergic neurons of the striatum (the caudate nucleus and putamen). This occurs in the presence of an order to execute a voluntary movement, which comes from the cerebral cortex and the SN pars compacta [23,24]. The degeneration of dopaminergic neurons in the SN pars compacta breaks the signalling pathway and deregulates the execution of voluntary movements, producing involuntary movements at rest that clearly define the symptoms present in PD patients [25].

The death of neurons in the substantia nigra is accompanied by the death of astrocytes (star-shaped glial cells) and an increase in the number of microglia cells in the substantia nigra. There are several reasons why the neuronal death of dopaminergic neurons can occur. One of the mechanisms is based on the accumulation of Lewy bodies, which are formed by the accumulation of insoluble proteins [26,27]. These accumulations can appear inside the neurons, forming the so-called Lewy body inclusions. Based on one of the theories of the pathological development of the disease, described by Heiko Braak, Lewy bodies first appear in the olfactory bulb, medulla oblongata, and pontine tegmentum [28]. Individuals with Lewy bodies in these brain structures are still asymptomatic or have early non-motor symptoms (sleep disturbances or a loss of the sense of smell) [29]. As Lewy bodies begin to appear in the SN, midbrain, forebrain, and neocortex, the development of motor and cognitive symptomatology becomes evident in PD patients [30,31].

The aim of this review was to describe the novel treatments for PD that are currently under investigation and study, as well as the possible therapeutic targets.

## 2. Results and Discussion

### 2.1. Pathophysiology of the Disease and Possible Therapeutic Targets

**Figure 1:**
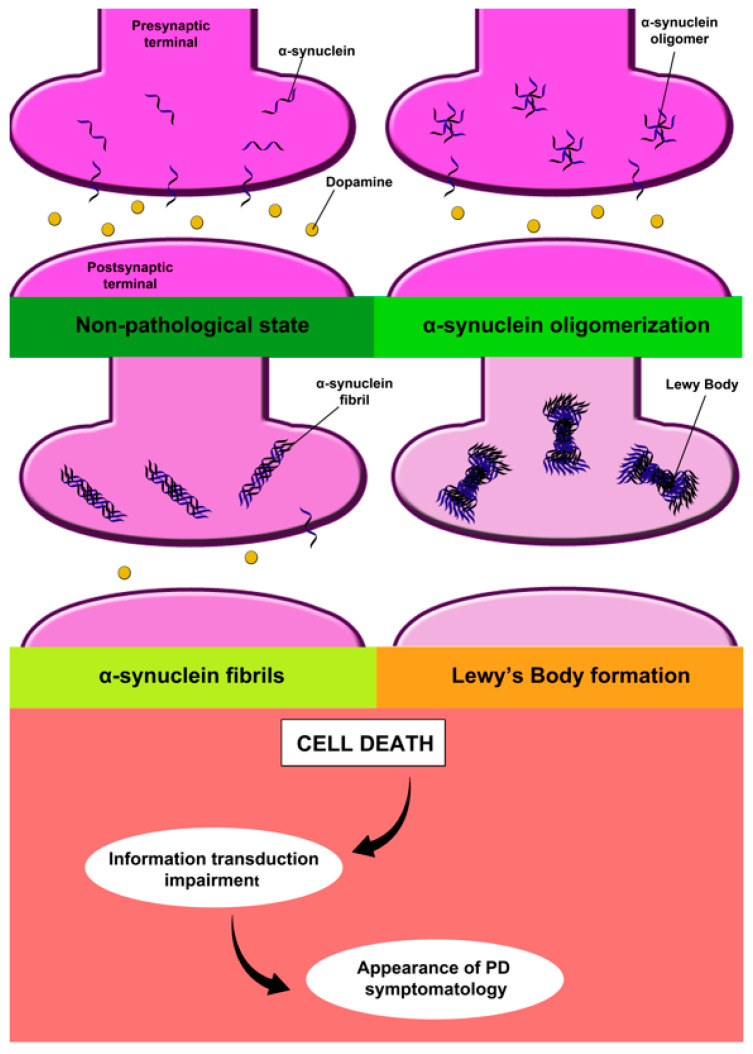
Pathophysiology of Parkinson’s disease.

Two pathophysiological processes can be clearly defined in PD: an alpha-synuclein abnormal folding accumulation that forms Lewy bodies and a malfunction of dopaminergic neurons in the substantia nigra pars compacta (SN), because alpha-synuclein (α-syn) becomes cytotoxic, damaging these cells. These processes cause the transduction of information in the brain to be altered, producing the symptoms of PD (Figure 1) [32]. Although the mechanism of dopaminergic neurons involved in neuronal death in PD has been described, the reason why this process is initiated is unknown. PD is defined by Lewy body disorder (LBD), which is based on the aggregation of alpha-synuclein [33]. It is accepted that alpha-synuclein aggregation can spread in a prion-like manner from neuron to neuron [34,35]. Nevertheless, it is still unclear where the onset of this pathology starts in PD. Some authors recently proposed two differential subtypes of PD: the body-first and brain-first models, which are based on where LBD originates [36,37,38]. In the body-first subtype, which is based on Braak’s hypothesis, alpha-synuclein aggregation starts in the enteric or peripheral nervous system and subsequently spreads to the CNS. In contrast, the “brain-first” subtype would start in the amygdala, followed by the substantia nigra and the locus coeruleus [39]. In addition, the symptoms and disease progression occur differentially in each variant. In the brain-first subtype, autonomic symptoms, sympathetic denervation, parasympathetic denervation, locus coeruleus degeneration, and REM sleep behaviour disorders occur after diagnosis, whereas in the body-first subtype this pathology appears in the prodromal stages. In addition, the progression of dementia and motor disabilities is more rapid in the body-first subtype than in the brain-first subtype [36,37,38]. This model differentiating two types of PD should be considered from a therapeutic point of view. The effectiveness of treatment may be higher in body PD as this group can be diagnosed before extensive brain damage occurs.

On the other hand, new theories are emerging on the possible involvement of the gut–brain axis in the pathogenesis of the disease. In this regard, different studies suggest a connection between the gut microbiota and brain that may impact neurodevelopment, brain performance, and overall health. It is believed that gut bacteria can affect PD through both indirect and direct pathways via the circulatory and nervous systems. The neural communication of the gastrointestinal tract involves multiple levels, starting with the myenteric submucosal plexus and enteric glial cells, with the catecholaminergic neurons being closest to the gut’s lumen [40]. The vagal nerve directly communicates with the myenteric plexus, connecting to prevertebral ganglia in the spinal cord and eventually to higher brain centres. The vagal afferent neurons located in the gut mucosa directly transmit information to the brain and have been shown to respond to LPS, a toxin produced by Gram-negative bacteria [41]. In PD, certain types of gut bacteria have been found to increase the production of inflammatory cytokines and LPS, resulting in damage to the intestinal epithelium and a compromised barrier [42]. This leads to increased gut permeability and the transfer of both bacterial toxins and host-derived inflammatory cytokines into the bloodstream, which can directly interact with the nervous system through a compromised blood–brain barrier (BBB) [43]. These cytokines are elevated in the sera of individuals with PD compared to healthy controls and may be linked to symptom severity and disease progression [44,45]. On this basis, several studies have explored the possible connection between α-synin-related pathology and the gastrointestinal symptoms of PD. In this regard, the German doctor Haiko Braak proposed the possible involvement of the GI tract in the development and progression of this pathology. Braak’s hypothesis states that pathogens can enter the body through the mouth, nose, or intestine and reach the GI tract, which could lead to the formation of Lewy bodies in cases of sporadic PD [46]. According to the hypothesis, Lewy bodies then form in the dorsal motor nucleus of the vagus in the medulla oblongata, the vagus nerves, and the anterior olfactory nucleus. Eventually, these Lewy bodies spread throughout the central nervous system and can be found in the substantia nigra, locus coeruleus, neocortex, mesocortex, and prefrontal cortex [47].

#### 2.1.1. Alpha-Synuclein

The α-syn protein is involved in synaptic maintenance: it regulates the size of dopamine vesicles in the presynaptic terminal, regulates the localization of dopamine transporters (DATs), and regulates dopamine synthesis [48]. It is expressed in several areas of the brain: the SN, hippocampus, neocortex, hypothalamus, and cerebellum [49]. The monomeric form of α-syn has a physiological role, but the poor folding and aggregation of α-syn monomers induces oligomers and fibres with a pathological character for neurons [50]. The change by which α-syn ceases to be monomeric and begins to aggregate by misfolding and making oligomers is unknown [51]. Although the role of α-syn in PD is known, the pharmacological treatments currently used do not act at the level of this protein.

Since the peripheral nervous system (PNS) is a target of α-synuclein deposition, there is a growing interest in assessing whether the intrinsic pathogenetic features of PD may predispose patients to peripheral neuropathy (PN). Recent studies have shown that PN occurs more frequently in individuals with PD compared to age-matched, healthy individuals. In this regard, there is growing evidence of pathological changes in the PNS of individuals with PD [52]. Deposits of α-synuclein have been found in the pharyngeal motor and sensory branch of the vagus nerve, the glossopharyngeal nerve, and the internal superior laryngeal nerve in PD patients who experience dysphagia [53]. Additionally, Lewy bodies have been discovered in the dorsal vagus ganglion, the parasympathetic sacral nuclei, the enteric nervous system, the cardiac plexus, and the pelvic plexus, even in early stages of the disease [54,55]. In addition to the findings mentioned above, there have been reports of α-synuclein aggregates in the unmyelinated fibres of the skin and of distal sensory and autonomic neuropathy in PD and other Lewy body disorders [56]. As we can find various markers at these levels that allow for the early detection of the pathology, this peripheral damage must be considered in the diagnosis of this disease.

#### 2.1.2. Dopamine

Dopamine is a neuromodulatory molecule that belongs to the catecholamine group of neurotransmitters. Dopamine is synthesized from tyrosine, which reaches the brain by an active transport mechanism. Tyrosine is generated by phenylalanine hydroxylase from phenylalanine in the liver. Once it is synthesized in the neuron, dopamine is included in the synaptic vesicles by vesicular monoamine transporter 2 (VMAT2) and is ready to be released into the synaptic cleft [57]. There are two families of receptors for dopamine, classified as D1 and D2. Subtypes 1 and 6 are included within the D1 family, and subtypes 2, 3, and 4 are included within the D2 family. All subtypes are coupled to a protein adenyl cyclase. The D1 subtype, which is abundant in the central nervous system in humans, is the most important dopaminergic receptor, followed by the D2 subtypes. The subtypes are all present in a multitude of brain structures; therefore, their roles differ depending on the structure in which they are expressed. 

Once dopamine has performed its function, it proceeds to its metabolism. There are two enzymes that metabolize dopamine: catechol-O-methyl transferase (COMT) and monoamine oxidase (MAO). Astrocytes contribute to dopamine metabolism as they express the MAO-B isoform, while the MAO-A isoform is present in catecholaminergic neurons. COMT is expressed mainly by glial cells. When dopamine levels are low in the brain, MAO synthesis decreases. Pharmacological targets of the dopaminergic system and/or dopamine metabolism are currently explored for treatment of PD [58,59]. 

**Figure 2:**
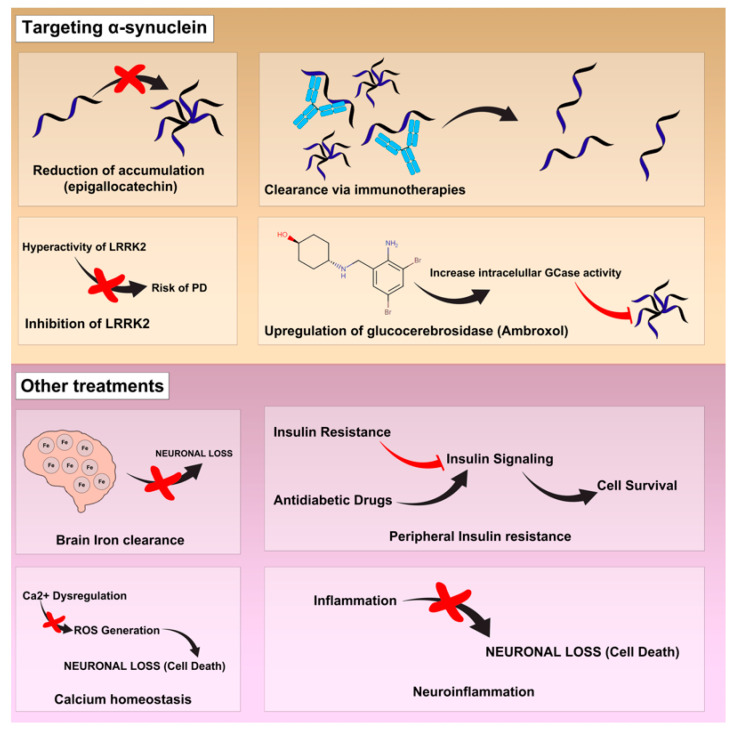
Current and emerging treatments of Parkinson’s disease.

To date, as previously mentioned, PD has no curative treatment or any therapy to modify the course of the disease. Thus, the purpose of its treatment is to alleviate the motor and non-motor symptoms that occur during the evolution of the disease [60,61]. Numerous treatments are currently available to treat the symptoms of this pathology. However, there are also other treatments, which are still under investigation to determine their therapeutic potential (Figure 2) [62].

### 2.2. Management of Motor Symptoms

The current pharmacological treatment is mainly based on the restoration of dopamine levels, with Levodopa considered the principal option [63,64,65]. This dopamine precursor was a breakthrough in the treatment of PD for reducing motor symptomatology and improving the quality of life of patients. It also led to the study of other dopaminergic therapies [66,67]. Nevertheless, the administration of Levodopa has limitations due to the occurrence of adverse reactions, with dyskinesia being one of the main complications [68]. Moreover, as the disease progresses, patients are less responsive to dopaminergic medication and require higher and more frequent doses of dopaminergics [51]. Therefore, current formulations of Levodopa contain decarboxylase inhibitors, better known as Carbidopa or Benserazide. The action of decarboxylase inhibitors is to prevent the peripheral metabolism of dopamine and allow for a greater bioavailability of the drug [69]. However, the simultaneous administration of Levodopa and other drugs is recommended in order to avoid the complications caused by high doses of a single medication [70]. These drugs include rasagiline, safinamide, selegiline, and Monoamine oxidase B (MAOB) inhibitors, which have been shown to increase dopamine levels [68]. Catechol-O-methyltransferase (COMT) inhibitors, such as entacapone and tolcapone, are also used. These tools stabilize dopamine levels and improve motor complications as they promotes the absorption of Levodopa at the gastrointestinal level, where most of this enzyme is found [69]. 

Another category of drugs are dopamine agonists such as ropinirole and pramipexole, which have been described as safe and effective in both monotherapy and in combination with Levodopa [71,72]. Within this category, the following drugs stand out: rotigotine, which is available in transdermal patches that provide a constant administration of the drug, [66] and apomorphine, which is used as a rescue treatment for patients with motor fluctuations through the administration of injections or subcutaneous infusions [73]. 

#### Invasive Treatment Options for Motor Symptoms

There are advanced treatments for patients with motor fluctuations or dyskinesias that lead to functional deterioration and do not improve despite the correct dosage of treatment [74]. Deep brain stimulation (DBS), the enteral suspension of Levodopa–carbidopa, and continuous subcutaneous infusion of apomorphine are among the best-known alternatives. DBS significantly reduces the off-time, obtaining a more beneficial effect on the patients’ quality of life compared to other therapies. In fact, the National Institute for Health and Care Excellence (NICE) guideline advises its application in the final stages of the disease [75]. Despite the need for a combination of dopaminergic treatments, there are cases in which the dose was reduced by 60% after its initiation [76]. Currently, DBS has been shown to be one of the most promising and safe alternatives in the treatment of PD. However, DBS is a strategy that requires surgery. Although it has been proven to be safe, surgery is not without risks, the most notable of which is the seizure associated with DBS implantation [77]. In addition, this treatment may have undesirable effects. Cases of patients with unwanted side effects such as symptoms of hypomania, mania, or even mania with psychotic symptoms after the use of deep brain stimulation have been described in the literature [78]. However, this technology has evolved significantly over the last 20 years, reducing unwanted side effects and more precisely targeting the areas to be stimulated according to the pathology to be treated [79]. Thus, DBS appears to be a solid therapeutic alternative under development for a number of neurological pathologies, including PD [80]

Alternatively, Levodopa–carbidopa enteral suspension is another surgical procedure that allows for the safe delivery of high doses of Levodopa by implanting a permanent tube through a percutaneous endoscopic gastrostomy (PEG) connected to a portable external pump. This treatment prevents fluctuations in Levodopa levels, which translates into less off-time and a lower risk of dyskinesias [81]. However, the occurrence of adverse reactions and high costs led to its abandonment by 34% of patients after 4 years [82]. 

Finally, the continuous subcutaneous infusion of apomorphine allows for the continuous administration of the drug and has the advantage of not requiring a surgical procedure. In addition, this type of treatment does not require high daily doses of Levodopa, and some cases have been described in which its administration was not necessary. However, one study found that after the first year, half of the patients ceased treatment due to a loss of efficacy and the appearance of adverse reactions [76]. 

### 2.3. Management of Nonmotor Symptoms

#### 2.3.1. Pharmacological Treatments

It is important to highlight that most PD treatments are mainly directed at motor symptomatology. However, PD is often accompanied by other known symptoms such as non-motor symptomatology. On one hand, we have neurological disorders related to the emotional impact that this disease has on the patients who suffer from it, such as apathy, anxiety, and depression, among others. On the other hand, we have symptoms related to the adverse effects of dopaminergic replacement, such as addiction or nocturnal hyperactivity. However, most of the non-motor symptoms do not respond to the aforementioned therapies and some even appear or worsen when these therapies are initiated [66]. It is quite common for these symptoms to occur in the early stages of the disease; therefore, their treatment is of vital importance. Another aspect to take into consideration is the fact that these symptoms occur even before the motor symptoms, suggesting that the non-motor symptoms are also due to a deficit of dopamine [83]. Nevertheless, the treatment of these symptoms is not specific to PD as they are similar to those used in the general population [74]. 

As previously mentioned, cognitive impairment, depression, sleep disorders, and autonomic dysfunction are the most common non-motor problems in PD [66]. Among the most commonly used drugs to treat cognitive impairment are acetylcholinesterase inhibitors such as Donepezil, Galantamine, and Rivastigmine [83]. For depression, Parkinson’s patients are often treated with serotonin/norepinephrine reuptake inhibitors (SNRIs) (e.g., Duloxetine, Desvenlafaxin, Milnacipran, and venlafaxine). Other therapeutical options are benzodiazepines (e.g., Alprazolam, Clonazepam, Diazepam, and Lorazepam), selective serotonin reuptake inhibitors (SSRI) (e.g., Fluoxetine and Sertraline), tricylic compounds (e.g., Amitrytiline, Imipramine, and Nortriptyline) and additional anxiolytics (e.g., Buspirone, Propanolol, Quetiapine, and Trazodone) [84]. With respect to sleep disorders, the affected patients often use Amitriptyline, Clonazepam, Doxepin, Eszopicione, Melatonin, Mirtazapine, and Trazadone. Nevertheless, it is necessary to consider cognitive behavioural therapy as a viable option to acquire habits for sleep hygiene, as well as to reduce anxiety levels and improve the prognosis of depression [85]. 

Although PD is known for its characteristic motor signs, Parkinson’s patients often experience a number of autonomic disorders such as gastrointestinal malfunction, cardiovascular dysregulation, urinary disturbance, sexual dysfunction, thermoregulatory aberrance, and pupillo-motor and tear abnormalities [86]. In this regard, increasing attention is being paid to the non-motor symptoms of PD, partly because they may be present at very early stages of the disease, sometimes years before the classic motor signs and symptoms become apparent [87]. A study by Chen et al. proposed that some of these autonomic symptoms, such as constipation, orthostatic hypotension, urinary dysfunction, erectile dysfunction, and pure autonomic failure, may be considered prodromal dysautonomic markers in PD prediction and diagnosis due to their early onset and high prevalence among these patients [88]. Thus, patients with orthostatic hypotension can be treated with Fludrocortisone, Pyridostigmine, and Droxidopa. Urinary incontinence can be palliated mainly with four types of drugs: Anticholinergics, such as Darifenacin, Oxybutynin, Solifenacin, and Tolterodine; Beta-3-Agonists, with the main option being Mirabegron; Alpha-1A blockers, among which Alfuzosin, Silodosin, Tamsulosin, and Terazosin stand out; and SNRIs such as Duloxetine. On the other hand, sialorrhea in Parkinson’s patients is usually due to a slowing of swallowing, which could be treated with Atropine drops, Botulinum toxins A and B, Glycopyrrolate, or a Scopolamine patch [89]. Finally, digestive problems such as constipation are usually addressed in the first instance with non-pharmacological measures such as dietary modification (e.g., consuming high-fibre foods and plenty of fluids). However, when this does not work, drugs such as Lubiprostone and Polyethylene glycol may be administered. For other conditions such as nausea and vomiting the most commonly used treatment options are ondansetron and trimethobenzamide [90].

#### 2.3.2. Non-Pharmacological Treatments

In the therapeutic approach to non-motor symptoms, there are non-pharmacological measures that should be considered Thus, it has been studied how the patients’ own management could improve cognitive impairment as a complement to the medication administered. These preliminary results show how aerobic exercise could improve cognitive functions in mild to moderate stages. In addition, the effect of cognitive training on dementia has been analysed, and an improvement has been observed in subjects who carried out these sessions with a frequency of two or three times per week in the following parameters: executive function, concentration, processing speed, and visual and working memory. However, it should be noted that, again, these interventions may have no effect on late-stage dementia; therefore, further research is needed on subjects with different stages of cognitive impairment [91,92,93].

Regarding sleep disorders, these alternatives are based on presenting an adequate sleep hygiene by limiting the consumption of exciting beverages such as coffee or tea at least 4 h before going to bed or avoiding naps, thus increasing activity during the day. Another alternative that has been shown to improve the regulation of the sleep–wake transition, thus improving sleep quality, is physical exercise. In addition, a significant benefit has been observed in physical activities in which a cognitive element is introduced, such as Tai Chi [94]. Cognitive behavioural therapy is one of the alternatives that has shown favourable results in the treatment of sleep disorders in PD. Among these therapies, light therapy stands out since it not only has a positive effect on regulating the sleep pattern but has also been shown to improve the mood and motor state of PD patients [95].

On the other hand, dietary habits induce certain modifications in the composition of the gut microbiota, which could improve the gastrointestinal symptoms associated with this pathology. For this reason, the administration of probiotics or live micro-organisms that have beneficial effects on health when ingested in adequate amounts is becoming increasingly important among the treatment options for PD [96]. The host’s gut microbiome can be altered by the use of probiotics through mechanisms such as competition for resources, inhibitory actions, and mutual support [97]. Probiotic bacteria can form biofilms, which increase their ability to colonise and persist in the intestinal mucosa while reducing the adhesion of harmful bacteria [98]. Many probiotic strains exhibit antimicrobial properties due to the secretion of organic acids, such as lactic acid, or bacteriocins, which suppress pathogens. In addition, probiotics and the host microbiota can collaborate to produce beneficial short-chain fatty acids such as butyrate [97]. The benefits of the administration of probiotics in PD have already been demonstrated. As previously mentioned, dysbiosis in the intestinal flora conditions the appearance of significant constipation in these patients, which often goes unnoticed and is not adequately treated. In this regard, Tan et al. conducted a randomised clinical trial among PD patients in which they administered a probiotic preparation based on *E. faecium*, *L. acidophilus*, *L. paracasei*, and *L. rhamnosus*, *B. longum*, *B. bifidum*, and *L. reuteri* or a placebo. The results of this study showed that the number of spontaneous bowel movements increased after treatment with the probiotic, which also improved the stool consistency, quality of life, and satisfaction among the patients who followed this treatment [99]. Du et al. carried out another randomised clinical trial based on the use of probiotics in PD patients. In this study, 46 patients with PD and constipation were divided into two groups: the intervention group, who received a probiotic treatment for 12 weeks that contained *Bacillus licheniformis*, *Lactobacillus acidophilus*, *Bifidobacterium longum*, and *Enterococcus faecalis*, and the control group, who continued to take their usual pharmacological treatment. It was observed that probiotic supplementation induced an improvement in the average number of complete bowel movements per week and reduced the Bristol stool scale score, the patient assessment of constipation symptom score, the patient assessment of constipation quality of life questionnaire score, and the degree of defecation effort score. A sequencing analysis of the bacterial flora after treatment showed that the species *g_Christensenella_sp._Marseille-P2437* significantly increased, while *g_Eubacterium_oxidoreducens_group*, *g_Eubacterium_hallii_group*, and *s_Odoribacter_sp._N54.MGS-14* decreased [100]. In addition to the favourable effects of probiotics on constipation, other authors observed improvements in diverse inflammatory markers such as IL-1, IL-8, TNF-α, TGF-β, and PPAR-γ following probiotic administration for 12 weeks in patients with PD [101]. Additionally, improvements in movement and various metabolic parameters were noted, as shown by Tamtaji et al. According to these authors, probiotic supplementation for 12 weeks improved the Movement Disorders Society-Unified Parkinson’s Disease Rating Scale and glutathione levels, reducing the high-sensitivity, C-reactive protein and malondialdehyde. Additionally, probiotic consumption resulted in a significant reduction in insulin levels and insulin resistance and a significant rise in insulin sensitivity [102].

### 2.4. Treatments under Investigation

The unavailability of treatment to reverse or prevent the progression of PD results in an unmet therapeutic need for patients, most of whom suffer from disabilities resulting from the multiple complications that occur during the course of the disease. Therefore, the search for a successful treatment is a major challenge for current research [103]. Recent studies on the genetic basis of PD have led to a better understanding of the pathophysiology of the disease, opening the path to new therapeutic targets and possible treatments, detailed below [104]. 

#### 2.4.1. Calcium Homeostasis

Dihydropyridine L-type calcium channel blockers used in the treatment of arterial hypertension appear to have a neuroprotective effect. This hypothesis has led to the study of several compounds including Isradipine, which was shown to be safe and tolerated in patients in the early stages of PD in a Phase II clinical trial [105]. In addition, a Phase III study was conducted to assess the efficacy of the treatment, although its results were able to confirm the role of calcium imbalance in the pathogenesis of PD due to a number of complications such as dosing problems, among others [106]. 

#### 2.4.2. Brain Iron Deposits

Iron accumulation at the cerebral level has been described as a typical feature in the post-mortem brains of Parkinson’s disease patients. Brain iron accumulation has been described as a typical feature in the post-mortem brains of Parkinson’s patients which could contribute to the loss of the substantia nigra [107]. Therefore, iron elimination has been studied as a possible therapeutic target. The use of deferiprone in a Phase II clinical trial was shown to improve motor symptomatology and reduce iron levels in the brain. These results reinforce the need for new clinical trials to study the neuroprotective effect of this compound [108]. 

#### 2.4.3. Peripheral Insulin Resistance

The discovery of Type II diabetes as a promoter of several neurodegenerative diseases, including PD, suggests an alternative in the search for a modifying treatment [109]. Oral antidiabetics, including exenatide, a glucagon-like peptide-1 (GLP-1) agonist, were administered in a Phase II clinical trial to patients with moderate PD. The Unified Parkinson’s Disease Rating Scale (UPDRS) score was found to be lower in the treatment group compared to the placebo group, showing a promising approach in the treatment of PD [105]. 

Dimethylbiguanide, better known as metformin, is an oral antidiabetic that has become highly relevant not only because of its proven efficacy in the regulation of glucose levels but also because it represents a promising approach in the treatment of neurodegenerative diseases. The latter could be explained by the fact that metformin has been shown to counteract the hypofunction of AMP-activated protein kinase (AMPK), which plays a crucial role in the maintenance of neuronal cells, the levels of which are decreased in the development of neurodegenerative diseases, including PD. However, it remains to be studied whether the administration of metformin in healthy patients could have a beneficial effect in age-related disorders such as PD [110]. 

#### 2.4.4. Neuroinflammation

Inflammatory processes at the brain level have been associated with PD as an important trigger of the disease [111]. In fact, a direct relationship has been observed between the inflammation occurring in the substantia nigra and the consequent dopaminergic neurodegeneration [112]. In addition, the NLRP3 inflammasome has been described as a key player in the neuroinflammation observed in neurodegenerative diseases such as PD [113]. Therefore, NLRP3 inhibitors such as Inzomelid could constitute a new therapeutic target [105]. Among NLRP3 inhibitors, MCC950 stands out. The administration of MCC950 in murine models of PD was found to reverse inflammasome activation, leading to an improvement in motor symptoms and a reduction in dopamine degeneration and α-synuclein deposits [114].

On the other hand, given the involvement of the Janus kinase/signal transducer and activator of transcription (JAK/STAT) cytokine signalling pathway in inflammatory processes, its inhibition has been studied as a possible therapeutic strategy aimed at the neuroinflammation occurring in PD [115]. In fact, the administration of AZD1480, a synthetic inhibitor, in a rodent model with overexpression of α-synuclein was shown to reduce microgliosis and macrophage infiltration in addition to decreasing levels of proinflammatory cytokines and MHC Class II expression [116].

#### 2.4.5. Pathology of α-Synuclein

The existence of a strong association between the neurotoxicity caused by α-synuclein oligomers and PD pathogenesis places the balance between α-synuclein production and clearance as a new therapeutic goal. Mechanisms involved in achieving α-synuclein homeostasis include decreasing its production, boosting its clearance, or preventing cell transmission [100]. Among the compounds studied, a polyphenol called epigallocatechin gallate has been shown to reduce α-synuclein accumulation and its neurotoxicity in a Phase II clinical trial. Despite good tolerance, the appearance of hepatic involvement led to a reduction in the dose administered [117]. Meanwhile, a number of heat shock proteins (Hsps) have been identified which act as chaperones, favouring the correct folding and aggregation of proteins such as α-synuclein [118]. An example of this is Hsp110, which has been shown to regulate α-synuclein transmission, thus exerting a neuroprotective effect [100]. A hopeful anti-aggregation treatment from Neuropore Therapies, UCB0599 (previously named NPT-200-11), has recently commenced a Phase II clinical trial (NCT04658186) involving 450 individuals in the early stages of PS. Its anticipated conclusion is in mid-2024 [119].

Another promising approach is to promote the clearance of and prevent the cellular spread of α-synuclein through immunotherapy [120]. There are several agents currently under study, including Cinpanemab and Prasinezumab, which are both used in passive immunotherapy. These compounds appeared to be safe and well tolerated in a Phase I clinical trial, which led to the development of Phase II clinical trials to assess efficacy [105]. However, the administration of both agents in Phase II was not effective, and adverse reactions were described after the use of Prasinezumab [62,121]. Of the compounds used in active immunotherapy, PD01A and PD03A stand out. Phase I clinical trials of both agents showed favourable results in terms of safety and tolerability. The administration of PD01A also resulted in stabilized clinical scores which, in combination with the above, prompted the continuation of this Phase II trial [122]. Moreover, a synthetic peptide developed by United Neuroscience (UB312) that mimics the oligomeric and fibrillar forms of α-synuclein is being evaluated in a Phase I clinical trial for PD (NCT04075318) [123]. Several other antibodies that target α-synuclein are currently in Phase I trials for passive immunotherapy, including MEDI1341 (AstraZeneca, Cambridge, UK), Lu-AF82422 (Lundbeck, Copenhagen, Denmark), ABBV-0805 (AbbVie, North Chicago, IL, USA/BioArctic, Stockholm, Sweden), ABL301 (Sanofi, Singapore/ABL Bio, Seongnam, Republic of Korea), and UCB7853 (UCB Biopharma, Brussels, Belgium/Novartis, Singapore) [124].

Meanwhile, after an increased risk of PD was observed following the administration of β-antagonists such as propranolol, β-adrenergic regulation was described as a possible therapeutic approach to targeting α-synuclein [125]. In fact, the use of β2-adrenergic receptor agonists (β2AR) resulted in a decrease in the α-synuclein gene (SNCA). Specifically, the administration of metaproterenol demonstrated a reduction in SNCA of more than 35% [126]. Other β2ARs were studied, including clenbuterol and salbutamol. The efficacy of clenbuterol in reducing the α-syn mRNA and protein levels, previously demonstrated in murine models, was further confirmed after achieving equilibrium in previously elevated α-synuclein levels in human SK-N-MC cells due to propranolol administration [127]. Another significant finding was the decrease in mRNA and α-syn protein levels in neurons following clenbuterol use in a subject with SNCA triplication, which is an atypical trigger of autosomal dominant PD [128]. 

Salbutamol trials were carried out in combination with Levodopa. They obtained favourable results as the effects lasted after the suspension of salbutamol and improved the motor symptoms derived from Levodopa treatment [127]. However, despite the efficacy demonstrated after the use of β2AR, there was an opposite effect in patients with IIDM, i.e., it triggered an increased risk of developing PD. This is explained by the influence of these agents on the stabilization of blood glucose levels as they intervene in insulin secretion, hepatic metabolism, and glucose uptake in the muscle [129].

It is also important to mention the role of leucine-rich repeat kinase 2 (LRRK2), as the hyperactivity of this kinase and the presence of mutations in it have been associated with an increased risk of PD. For this reason, the study of LRRK2 inhibition as a potential treatment has been proposed [130]. One of the LRRK2 inhibitors recently studied in Phase I and Ib clinical trials is DNL201. This compound has been shown to have the ability to stabilize lysosomal function in PD patients, demonstrating adequate safety and tolerability parameters [131]. 

Finally, the discovery of the relationship between the presence of mutations in GBA, the gene encoding glucocerebrosidase (GCase), and the subsequent risk of developing PD has provided an alternative in the study of new therapeutic strategies [132]. The mechanism by which these treatments could modify the course of PD is based on driving an increase in GCase levels that reduces the accumulation of α-synuclein. Ambroxol, which is known for its use as an antitussive, stands out among the agents studied. This drug was shown to increase GCase concentration while exerting an opposite effect on α-synuclein levels in both cellular and in vivo models [105]. Due to the satisfactory results with Ambroxol, this drug was studied in a Phase II clinical trial [133]. Latrepirdine, another macroautophagy-enhancing agent, underwent Phase III clinical trials for the treatment of Alzheimer’s disease. It was found to be well-tolerated with no significant toxicity. However, the trials showed that it was not more effective than a placebo (NCT00912288; NCT00838110; NCT00675623; NCT00954590). Despite early indications that latrepirdine might act primarily on alpha-syn, it likely affects many cellular processes, and its exact biochemical mechanisms are yet to be fully understood. 

#### 2.4.6. Faecal Microbiota Transplantation

The gut microbiota acts as a modulator of the nervous system and can directly or indirectly influence the biochemistry of neurons [134]. In this regard, recent research has shown that a dysbiosis of the gut flora can drive the growth of bacteria that may contribute to the accumulation of α-synuclein in the gut, and that this protein can then migrate to the central nervous system, providing a possible mechanism for the development of sporadic PD [135].

It is now known that in PD, patients suffer from gastrointestinal dysfunction due to local bacterial overgrowth. The intestinal dysbiosis that occurs in PD is a consequence of a decrease in intestinal biodiversity with a predominance and overgrowth of certain species that are detrimental to the organism. Thus, a particular increase in the number of Bacteroides has been observed in addition to a significant decrease in the genus Faecalibacterium, microorganisms that are very benign as they produce butyrate with an anti-inflammatory effect [136]. In the same vein, Qian et al. found a dysbiosis of the gut flora in PD patients, manifested by an overgrowth of the genera Clostridium IV, Aquabacterium, Holdemania, Sphingomonas, Clostridium XVIII, Butyricicoccus, and Anaerotruncus, while the genera Escherichia/Shigella were negatively associated with the duration of the disease. Due to these changes in flora, Yu et al. found that those predisposed to PD often suffered from recurrent constipation many years before the onset of the disease [137]. Therefore, and in view of the importance of bacterial composition in this disease, faecal microbiota transplants (FMT) are proving to be a promising therapeutic approach in the management of this pathology. This treatment consists of obtaining faeces from healthy donors, subjecting the faeces to a purification procedure, and then using the faeces to inoculate different sections of the intestine of people with Parkinson’s disease, mainly the terminal ileum, cecum, and colon [138]. Recent work using mice in which PD was induced demonstrated that intestinal dysbiosis in the animals was responsible for Parkinsonian symptoms and changes in the rodents’ normal behaviour [139]. In one study, the faecal microbiota of mice were analysed by RNA sequencing prior to the induction of PD, after the onset of the disease, and after being subjected to FMT using faeces from healthy mice. The altered gut microbiota was found to be manifested by a large increase in the number of bacteria producing short-chain fatty acids and toxins, both of which are responsible for inflammation of the intestinal mucosa and subsequent neuronal damage [140]. Zhong et al. concluded that the data from the stool analysis after FMT indicated that the treatment was effective in correcting gut microbiota dysbiosis, providing a protective effect on dopaminergic neurons and improving motor function [141]. Similar studies in humans corroborated the results previously reported in animals. Some authors evaluated the efficacy of FMT by analysing motor and non-motor symptoms such as quality of life, sleep time, constipation, depression, stress, and anxiety, observing a considerable improvement in each of these symptoms [134,142]. Based on these results, it can be considered that TMF biotherapy could be effective in PD patients because it regenerates the intestinal microbiota, preventing the formation of substances that cause inflammation of the enteric mucosa and affect both systemic and neuronal levels through the intestinal microbiota–enteric system–brain axis [139]. 

#### 2.4.7. Neurotrophic Factor Supplementation

Since one of the triggers of PD is the deterioration of dopaminergic neurons and the accumulation of Lewy bodies in the SN, the maintenance of this cell population has become a therapeutic target. Thus, neurotrophic factors (NTF) have been proposed as a tool to enhance the neuroprotection of this cell population. NTFs are peptides whose role is to enhance neuronal survival by improving the growth, maturation, and survival of multiple neuronal cell populations and favouring their regeneration after injury [143]. In this regard, numerous NTFs have been investigated for the treatment of PD, including mesencephalic astrocyte-derived NTF (MANF), brain-derived neurotrophic factor (BDNF), dopaminergic cell NTF (CDNF), glial cell NTF (GDNF), neurotrophin-3 (NT-3) and neurotrophin-4 (NT-4) [144].

In in vitro trials, some of these factors have shown great benefits in improving the survival of dopaminergic neurons and decreasing apoptosis, inflammation, and the stress suffered by the endoplasmic reticulum [145]. However, a handicap for the application of this therapy in clinical trials is the difficulty NTFs have in crossing the BBB, needing to be administered through a surgical route. On the other hand, the trials that tested the effect of this intervention demonstrated moderate and diverse effects in patients with PD [146]. Although improvements in motor symptoms and in the quality of life of patients have been observed in some cases, these benefits disappeared after a few months of NFT application, and patients needed an even higher dose of drugs for the treatment of their symptoms. In addition to the limited effects observed in some of the trials, the risks associated with a technique as invasive as intracranial infusion must be taken into account as there may be a greater probability of infection in patients treated with this technique [147]. 

However, the administration of NTFs by a less-invasive route is also being investigated, providing a promising approach with fewer adverse effects. Thus, recombinant adeno-associated viruses (AAV) have been tested as a vehicle in different neurodegenerative diseases, including PD. In in vivo assays, their administration has been associated with improved neuronal performance and an increased density of the fibres that compose the striatum [148]. Although these results are encouraging, it is necessary to carry out a greater number of trials and to transfer to clinical trials the factors that have shown greater potential in in vitro and in vivo assays.

#### 2.4.8. Stem Cells Transplantation

Over the last decades, the field of cellular genetics has undergone significant progress. This has generated new approaches in the study of stem cells with the aim of modifying the course of neurodegenerative diseases such as PD [149].

The numerous limitations derived from traditional cell therapies motivated the development of new techniques based on cell reprogramming and personalized medicine. Specifically, advances in the field of therapeutic innovation arose as a consequence of the ethical–political obstacles and difficulties associated with the search for donors in the use of embryos for the procurement of embryonic stem cells (ESCs) [150].

In this regard, one of the strategies that has shown promise is the use of induced pluripotent stem cells (iPSCs) [151]. iPSCs have a number of advantages that can compensate for the limitations associated with the use of ESCs, the most notable of which is the absence of ethical conflicts due to the fact that they do not use human embryos. Likewise, iPSC strategies have proven to be safer and to reduce the risk of immunological rejection since they have the possibility of being generated from specific cells of the patient through a less-invasive method [152]. Currently, iPSCs have been used in clinical trials with non-human primate models and have shown significant improvements. They have been shown to increase cell survival and generate a lower immune response from lymphocytes and microglia in the presence of histocompatibility. In addition, a remarkable survival of dopaminergic neurons has been observed in the post-mortem analysis of brain tissue after iPSC transplantation [153].

However, despite having demonstrated optimal results in non-human primate models, the efficacy of iPSCs in PD patients is still unknown. In this regard, it is important to note that the first clinical trial (JMA-IIA00384, UMIN000033564) was initiated in Japan to treat patients with moderate PD using iPSC-derived dopaminergic progenitors, the results of which are still unknown [154]. Moreover, there are still many questions and ethical issues to be resolved regarding cell therapies. However, the study of iPSCs could provide a new approach to therapeutic alternatives in PD [153].

### 2.5. Supplementary Therapies for Treatment and Prevention of PD

#### 2.5.1. Music Therapy

Music therapy is defined as the use of sound and music within an evolving patient–therapist relationship to support and develop physical, mental, spiritual, and social well-being [155]. Music therapy is an inexpensive and non-invasive therapy, the main advantage of which is the absence of the side-effects of pharmacological therapy [156]. This type of treatment has been shown to be effective in a variety of neurodegenerative pathologies such as AD, Huntington’s disease, and PD [157]. In relation to the latter, music therapy has been shown to be an effective intervention in the therapeutic approach to this pathology by improving the motor function, balance, freezing of gait, gait speed, and mental health of affected patients [158]. If we focus on motor benefits, recent clinical trials have shown that therapies based on auditory rhythmic stimulation or singing are able to improve gait-related parameters, reduce the risk of falls, improve general motor performance, or even compensate for the loss of automatic and rhythmic movements [159,160]. Similarly, auditory rhythms reinforce the stability of movement in time and space [161]. However, not all types of music produce the same benefits in patients with PD, and the desired effect of this therapy must be studied. Thus, classical music has been shown to help reduce trunk velocity and trunk tilt, while genres such as rock or heavy metal improve the range of pelvic obliquity movements in the frontal plane [162]. Finally, it should be noted that music therapy not only has favourable effects in the motor sphere: its benefits are also transferred to other areas such as communication, emotional management, and cognitive areas [163].

#### 2.5.2. Diet

A healthy diet can play a protective role against PD by delaying the onset of the symptoms that characterise PD [164]. In this regard, diets that are rich in vegetables with a moderate intake of unsaturated fatty acids and a low intake of saturated fatty acids have been found to protect against the onset of both motor and non-motor symptoms of Parkinson’s disease [165]. This type of dietary pattern provides a large amount of antioxidant and anti-inflammatory compounds that promote the protection of the enteric and nervous system by preventing neuroinflammation, oxidative stress, and the accumulation of α-synuclein, which is responsible for the death of dopaminergic neurons [164]. In this sense, the Mediterranean diet meets these characteristics as it is based on the consumption of fruit, vegetables, legumes, cereals, olive oil, and fish, while red meat, poultry, and dairy products are consumed in moderation [166]. Several studies have shown a reduced likelihood of suffering from Parkinson’s symptoms and even a deceleration in the development of these symptoms at an early stage of the disease thanks to the Mediterranean diet [165]. A variant of this pattern is known as MIND (Mediterranean-DASH Intervention Neurodegenerative Delay), which results from a combination of the Mediterranean Diet and the DASH (Dietary Approaches to Stop Hypertension) diet. Adherence to this diet delays the age of onset of PD by an average of 17 years for women and 8 years for men, improving the results obtained for the Mediterranean diet [167]. It seems that the success of the dietary pattern lies in the increased consumption of foods that provide nutrients with a protective effect on neurons against the deterioration that comes with ageing [168]. Some of these foods are green leafy vegetables and berries that contain high amounts of carotenes, vitamin B9, and vitamin E, which have important anti-inflammatory and antioxidant properties that directly affect neuronal health in a beneficial way [169]. 

#### 2.5.3. Phytotherapy

Phytotherapy is characterised by the use of compounds of natural origin present in various plant species for therapeutic purposes [170]. In the case of PD, some bioactive compounds have also been identified that could have a positive impact on the course of this disease. Thus, there is evidence demonstrating the antioxidant, anti-inflammatory, and therefore neuroprotective role of different herbs present in traditional Chinese and Indian medicine [171]. These effects are extensible to other types of molecules, such as the phenolic compounds present in many nutritional products (e.g., secoiridoids, phenolic alcohols, phenolic acids, flavonoids, and lignans). In this sense, multiple in vivo assays have evidenced a decrease in neuroinflammation, a control of cellular iron, a regulation of signalling pathways, and a palliation of proteinopathy, among others. These results were accompanied by improvements in behavioural tests in which benefits were observed in locomotor activity, turning time, and motor balance as well as a decrease in bradykinesia [172]. In addition, it should be noted that these compounds are capable of crossing the blood–brain barrier. Thus, in vitro tests with brain endothelial cells demonstrated that some flavonoids are able to penetrate the blood–brain barrier [173]. Similar results have been obtained in in vitro assays with these same compounds: it was observed that after dietary intake, flavonoids were localizable in the brain tissue of the studied animals [174]. These are all promising results regarding the management of the pathophysiology of PD; however, further studies are needed to explore the therapeutic utility of these compounds in order to translate them into clinical practice.

### 2.6. Main Handicaps in the Treatment of Parkinson’s Disease

The difficulty in diagnosing PD is one of the main obstacles to its treatment. PD is diagnosed when clinical symptoms are present. As previously mentioned, not all PD patients show overt symptoms, at least in the early stages of the disease [11]. In addition, PD can produce symptoms compatible with dementia. In this sense, the distinct dementia produced by PD can be confused with the development of Alzheimer’s disease. It has even been proposed that the combination of Lewy pathology and Alzheimer’s disease pathology (β-amyloid plaques and neurofibrillary tangles) is the strongest pathological correlate of dementia in PD [175]. Indeed, the parieto-occipito-temporal junction is a prominent site of cortical pathology in PD-induced dementias, correlating significantly with cognitive impairment [176,177]. The controversial pathophysiological correlates of both diseases have prompted numerous clinical trials to test combination therapies against the pathological molecules of both diseases (PD and Alzheimer’s). In particular, antibodies against α-synuclein, tau, and beta-amyloid are being tested with modest results [178,179].

On the other hand, another limitation in the therapeutic approach to PD is the difficulty, on many occasions, in making the developed drugs reach the dopaminergic neurons in the nigrostriatal area. The blood-brain barrier filters the access of molecules to the brain, allowing the passage of molecules that are smaller or have a specific entry channel [180]. This phenomenon limits the pharmacological options useful in the treatment of neurological diseases. Numerous lines of research are currently focused on improving the permeability of this barrier to allow the entry of molecules that cannot naturally access the CNS [181,182,183]. In this sense, using forced ultrasound to open the blood–brain barrier to deliver viral vectors against alpha-synuclein and improve the delivery of neurotrophic proteins has shown reductions in PD-associated pathology in experimental models, although the results of this work have been modest [184,185]. Recently, prospective Phase I clinical trials in humans involving the opening of the blood–brain barrier with focused ultrasound have, thus far, demonstrated that this technique is safe and provides quantitative benefits in the treatment of PD [186,187]. If these preclinical and clinical studies confirm the usefulness of this technique, it would be a fundamental paradigm shift in the treatment of CNS diseases.

Despite advances in the therapeutic approach to PD, we still face significant challenges in the area of treatment development and evaluation. One of the main obstacles limiting the control of this pathology is the lack of more complete animal models that include important factors, such as ageing and peripheral pathology, which may also condition the low translational value of preclinical results [188]. Thus, clinical trials testing drugs for their ability to modify PD progression have shown mixed results. These results could be attributed to different factors, such as the diverse nature of the pathophysiology and clinical presentation of PD, the difficulty in identifying PD in its premotor phase, and the insufficiency of objective and reliable results to assess drug efficacy [189]. Furthermore, we can currently only measure the impact of the disease through clinical markers (motor and non-motor) and indirect markers of degeneration (such as functional imaging) which are the result of neuronal deterioration. However, these markers may not accurately reflect disease status, progression, or response to treatments as they are not directly related to the disease process. In addition, these markers may be affected by factors unrelated to the neurodegenerative process, such as the side effects of experimental interventions, which may lead to misinterpretation of these markers as a modification of the disease. Similarly, patients in clinical trials often receive dopaminergic medication which has important symptomatic effects, making it difficult to discern disease-modifying effects from other treatments. These limitations have made it difficult to conduct studies aimed at demonstrating disease modification [190].

## 3. Conclusions

Parkinson’s disease continues to be a pathology of unknown origin that generates a significant social cost for the patients who suffer from it. Although there is still no definitive cure for this disease at present, there are numerous treatments available aimed at reducing the symptomatology of PD, in addition to other therapeutic alternatives that are still under investigation. However, the therapeutic approach to this pathology should include a combination of pharmacological and non-pharmacological strategies to maximise outcomes and improve symptomatological control in these patients. Finally, a thorough understanding of the pathophysiology underlying this disease is necessary to be able to offer treatments that improve the quality of life of patients with minimal adverse effects.

## Data Availability

Data sharing not applicable.
